# Supplementary material for: Availability of results of interventional studies assessing colorectal cancer from 2013 to 2020
Source: PLoS One. 2022 Apr 11;17(4):e0266496. doi: 10.1371/journal.pone.0266496 (PMC9000106; doi:10.1371/journal.pone.0266496)
Supplement: S1 Table — (DOCX) [file pone.0266496.s001.docx]

**S1 Table.** Advanced search on ClinicalTrials.gov with the advanced research tool.

| **Used search fields** |  |
| --- | --- |
| Condition or disease | Colorectal cancer, colorectal neoplasms |
| Study type | Interventional studies (clinical trials) |
| Status | Completed, terminated |
| Sex | All |
| Study population | Adult, Older Adult (65 and over) |
| Primary completion | From 01/01/2013 to 01/01/2020 |
| Results | With and without study results |
