# Supplementary material for: Availability of results of interventional studies assessing colorectal cancer from 2013 to 2020
Source: PLoS One. 2022 Apr 11;17(4):e0266496. doi: 10.1371/journal.pone.0266496 (PMC9000106; doi:10.1371/journal.pone.0266496)
Supplement: S2 Table — (DOCX) [file pone.0266496.s002.docx]

**S2 Table.** Data extraction form.

| **ClinicalTrials.gov identifier (NCT number)** |  |
| --- | --- |
| **Official title of the interventional study** |  |
| **Acronym of title** |  |
| **Phase** |  |
| **Start date** *(Month Day, Year)* |  |
| **Primary Completion date** *(Month Day, Year)* |  |
| **First posted date** *(Month Day, Year)* |  |
| **Last update date** *(Month Day, Year)* |  |
| **Results posted on ClinicalTrials.gov** | Has results  No results |
| **Results first posted date** *(Month Day, Year)* |  |
| **Provided documents** *(study documentation, Study protocol, Statistical analysis plan, Informed consent form)* | Yes  No |
| **Sponsor/Collaborators (funding)** |  |
| **Funded by** | Industry  Non-industry  Both |
| **Individual patient data (IPD) sharing statement** | Yes  No  Undecided  NA |
| **Description of IPD sharing statement** |  |
| **Condition(s)** *(as mentioned in the registry record)* |  |
| **Number of arms** |  |
| **Gender** | Male  Female  All |
| **Age of enrollment** | Minimum: Maximum: |
| **Location** *(countries)* |  |
| **Enrollment** *(number)* |  |
| **Type of enrollment** | Planned/Estimated  Actual |
| **Study design** *(intervention model)* | Allocation:  Intervention model:  Masking (Blinding):  Primary purpose: |
| **Type(s) of intervention(s) and name(s)** *(put all interventions if more than one)* |  |
| **Type(s) of outcome measure(s)**  *(list all)* | Current primary outcome(s)  Current secondary outcome(s) |
| **Link for publication of results available in registry** | Yes  No |
