# Supplementary material for: Availability of results of interventional studies assessing colorectal cancer from 2013 to 2020
Source: PLoS One. 2022 Apr 11;17(4):e0266496. doi: 10.1371/journal.pone.0266496 (PMC9000106; doi:10.1371/journal.pone.0266496)
Supplement: Attachment — Submitted filename: Response to reviewer results.docx [file pone.0266496.s004.docx]

March 17th, 2022

Dr Anna Pellat

Service de Gastroentérologie et d’Oncologie digestive, Hôpital Cochin, APHP

27 rue du Faubourg Saint Jacques 75014 PARIS

Tel: +33 (6) 89851724

annapellat@gmail.com

M.: PONE-D-21-34103

Title: “Availability of results of interventional studies assessing colorectal cancer over the past seven years.”

***Editor comments:***

***Dear Dr. Pellat,

Thank you for submitting your manuscript to PLOS ONE. After careful consideration, we feel that it has merit but does not fully meet PLOS ONE’s publication criteria as it currently stands. Therefore, we invite you to submit a revised version of the manuscript that addresses the points raised during the review process.***

Dear Editors,

We thank you and the two anonymous referees for the detailed and constructive comments on our revised manuscript entitled “Availability of results of interventional studies assessing colorectal cancer over the past seven years” by Pellat *et al.* Please find enclosed a new revised version that we submit for publication that takes these new comments into account. You will find below point-by-point responses to the comments and description of the changes made in the manuscript.

Thank you for your attention on our new revised manuscript.

Sincerely yours,

Ms. Anna Pellat

**Reviewer comments:**

***Reviewer #1***

*No comments*

***Reviewer #2***

*The authors adequately addressed most of my prior comments. A few new (based on revisions) and remaining issues are noted here:*

***Comment 1:**
*- Recommend revising the manuscript title to replace “over the past 7 years” to specify the actual years included in the analysis*

**Response 1:**

Thank you, we have made the requested change in the new revised version of the manuscript.

Title: “Availability of results of interventional studies assessing colorectal cancer from 2013 to 2020.”

***Comment 2:**

*- Lines 99 – 101 – it could be clarified that these are response options for the ‘primary purpose’ data element and the ‘intervention type’ data elements.*

**Response 2:**

Thank you for the suggestion. We have made the change as follows:

Modification in the methods section, line 99:

“We defined as management all trials with a primary purpose of screening, diagnosis, prevention (secondary and tertiary), treatment (including drugs, dietary supplement, behavioral intervention, device and surgery), and supportive care. Of note, these are response options proposed by ClinialTrials.gov for primary purpose and treatment/intervention types.”

***Comment 3:**

*- Consider if the explanation of ‘open-access’ be instead included in the ‘eligibility of results’ section (i.e., did the reviewer as a third step note if the published results were open access?)*

**Response 3:**

We agree with the reviewer and have made the following changes in the manuscript:

Modification in the methods section, “eligibility criteria for results section”, line 150:

“We considered results to be available in open access when available on ClinicalTrials.gov and/or in an open access publication downloaded from our institution.”

***Comment 4:**

*- Lines 158 – 159 – recommend specifying which data elements determine funding (was it sponsor and collaborators?)*

**Response 4:**

Thank you for this comment. Indeed, type of funding is determined by the “sponsor and collaborator” item from clinicaltrials.gov. We have made the following change in the manuscript, as well as in supplementary table 2:

Modification in the methods section, line 158:

“Type of funding (sponsors and collaborators) was divided in three categories”

***Comment 5:**

*- Table 2 – at a minimum denominator (# trials) needs to be added to the table column/row headers to provide appropriate context to the percentages, but could provide clarity by including numerator and denominator for each percentage*

**Response 5:**

We agree with the reviewer and have added the denominators for each row in Table 2.

***Comment 6:**

*- Line 291 – appears to describe the results from Table 3 re: randomized trials but the data are different. Please verify (HR=1.2 (95%CI 0.9 – 1.6)) v. (HR=1.0 (95% CI 0.8 -1.4)).*

**Response 6:**

Thank you for this comment. Table 3 only shows the results of the post-hoc multivariate analysis (after reviewing). In the text, we have left the results for the initial univariate analysis which does not appear in Table 3 (this explains the different HR values).

***Comment 7:**

*- I don’t agree that the example of results before PCD demonstrates lack of currency or quality (Lines 358) but appreciate the authors qualification of a potential reason for this particular event*

**Response 7:**

Thank you for your understanding and for accepting this explanation in the discussion.

***Comment 8:**

*- Line 349 – misquotes the law and should be revised from "applicable trials" to “applicable clinical trials"*

**Response 8:**

Thank you for this remark. We have made the correction in the revised version of the manuscript
